# Supplementary material for: Heat adaptation of phage T7 under an extended genetic code
Source: Virus Evol. 2021 Dec 1;7(2):veab100. doi: 10.1093/ve/veab100 (PMC8923235; doi:10.1093/ve/veab100)
Supplement: veab100_Supp [file veab100_supp.zip › Sequencing_Analysis.zip!/Sequencing_Analysis/S3/evidence/JC_SIDE_2_52.html]

BRESEQ :: Evidence


| New junction evidence | | | | | | | | | | | |
| --- | --- | --- | --- | --- | --- | --- | --- | --- | --- | --- | --- |
|  | | seq id | position | reads (cov) | reads (cov) | score | skew | freq | annotation | gene | product |
| \* | ? | NC\_001604.1 | = 16508 | 832 (0.480) | 277 (0.160) | 147/586 | 6.7 | 27.7% | intergenic (–/–) | *–/–* | –/– |
|  | ? | NC\_001604.1 | 16970 = | 637 (0.380) |  |  |  |  | intergenic (–/–) | *–/–* | –/– |
| Rejected: Coverage evenness skew score above cutoff. | | | | | | | | | | | |

Only 100 of 673 total aligned reads displayed.  

`TGCCAAGCAGGTTAGGCTAGGTGGATGCGGTAGGTCCGAATATAAGGATGGAGACTTTGACATTCTTGCGGTTGTGGTTGACGAAGATGTGCTTATTTTCACATGGGACGAAGTAAAAGGTAAGACATCCATGTGTGTCGGCAAGAGAAACAAAGGCATAAAACTATAGGAGAAATTATTATGGCTATGACAAAGAAATTTAAAGTGTCCTTCGACGTTACCGCAAAGATGTCGTCTGACGTTCAGGCAATCTTAGAGAAAGATATGCTGCATCTATGTAAGCAGGTCGGCTCAGGTGCGATTGTCCCCAATGGTAAACAGAAGGAAATGATTGTCCAGTTCCTGACACACGGTATGGAAGGATTGATGACATTCGTAGTACGTACATCATTTCGTGAGGCCATTAAGGACATGCACGAAGAGTATGCAGATAAGGACTCTTTCAAACAATCTCCTGCAACAGTACGGGAGGTGTTCTGA‑TGTCTGACTACCTGAAAGTGCTGCAAGCAATCAAAAGTTGCCCTAAGACTTTCCAGTCCAACTATGTACGGAACAATGCGAGCCTCGTAGCGGAGGCCGCTTCCCGTGGTCACATCTCG  >  NC_001604.1/16671‑17269`  

                                                                                                                                                                                                                                                                                                           `|`                                                                                                                                                                                                                                                                                                              
 `gCCAAGTAGGTTAGGCTAGGTGGATGCGGTAGGTCCGAATATAAGGATGGAGACTTTGACATTCTTGCGGTTGTGGTTGACGAAGATGTGCTTATTTTCACATGGGACGAAGTAAAAGGTAAGACATCCATGTGTGTCGGCAAGAGAAACAAAGGCATAAAACTATAGGAGAAATTATTATGGCTATGACAAAGAAATTTAAAGTGTCCTTCGACGTTACCGCAAAGATGTCGTCTGACGTTCAGGCAATCTTAGAGAAAGATATGCTGCATCTATGTAAGCAGGTCGGCTCAGGTGCGAt                                                                                                                                                                                                                                                                                                            >  1:254763/1‑301 (MQ=255)`  
  `ccAAGCAGGTTAGGCTAGGTGGATGCGGTAGGTCCGAATATAAGGATGGAGACTTTGACATTCTTGCGGTTGTGGTTGACGAAGATGTGCTTATTTTCACATGGGACGAAGTAAAAGGTAAGACATCCATGTGTGTCGGCAAGAGAAACAAAGGCATAAAACTATAGGAGAAATTATTATGGCTATGACAAAGAAATTTAAAGTGTCCTTCGACGTTACCGCAAAGATGTCGTCTGACGTTCAGGCAATCTTAGAGAAAGATATGCTGCATCTATGTAAGCAGGTCGGCTCAGGTGCGAtt                                                                                                                                                                                                                                                                                                           >  1:313220/1‑301 (MQ=255)`  
   `atacagggTTAGGCTAGGTGGATGCGGTAGGTCCTGATATAAGGATGGAAGATTTGACATTTTTGCGGTTGTGGTAGATGAATATGTGCTTATTTTCACATGGGACGAAGTAAAATGTAAGACATCCATGTGTGTCCGCAAGAGAAACAAATGCATAAAACTATAGGAGAAATTATTATTGCTATGACAAAGAAATTTAAAGTTTCCTTCGACGTTACCGCAAAGATGTCGTCTGACGTTCAGGCAATCTTAGAGAAAGATATGCTGCATCTATGTAAGCAGGTCGGCTCAGGTGCGATTg                                                                                                                                                                                                                                                                                                          <  1:338717/295‑1 (MQ=255)`  
         `ggTTAGGCTAGGTGGATGCGGTAGGTCCGAATATAAGGATGGAGACTTTGACATTCTTGCGGTTGTGGTTGACGAAGATGTGCTTATTTTCACATGGGACGAAGTAAAAGGTAAGACATCCATGTGTGTCGGCAAGAAAAACAAAGGCATAAAACTATAGGAGAAATTATTATGGCTATGACAAAGAAATTTAAAGTGTCCTTCGACGTTACTGCAAAGATGTCGTCTGACGTTCAGGCAATCTTAGAGAAAGATATGCTGCATCTATGTCAGCAGGTCGGCTCAGGTGCGATTGTccccc                                                                                                                                                                                                                                                                                                    >  1:148892/1‑300 (MQ=255)`  
                  `aGGTGGATGCGGTAGGTCCGAATATAAGGATGGAGACTTTGACATTCTTGCGGTTGTGGTTGACGAAGATGTGCTTATTTTCACATGGGACGAAGTAAAAGGTAAGACATCCATGTGTGTCGGCAAGAGAAACAAAGGCATAAAACTATAGGAGAAATTATTATGGCTATGACAAAGAAATTTAAAGTGTCCTTCGACGTTACCGCAAAGATGTCGTCTGACGTTCAGGCAATCTTAGAGAAAGATATGCTGCATCTATGTAAGCAGGTCGGCTCAGGTGCGATTGTCCCCAATGGTAAAc                                                                                                                                                                                                                                                                                           >  1:242872/1‑301 (MQ=255)`  
                    `gTGGAGGCGGTAGGTCCGAATATAAGGGTGGAGACTTTGACATTCTTGCGGTTGTGGTTGACGAAGATGTGCTTATTTTCACATGGGACGAAGTAAAAGGTAAGACATCCATGTGTGTCGGCAAGAGAAACAAAGGCATAAAACTATAGGAGAAATTATTATGGCTATGACAAAGAAATTTAAAGTGTCCTTCGACGTTACCGCAAAGATGTCGTCTGACGTTCAGGCAATCTTAGAGAAAGATATGCTGCATCTATGTAAGCAGGTCGGCTCAGGTGCGATTGTCCCCAATGGTAAACAg                                                                                                                                                                                                                                                                                         <  1:232534/301‑1 (MQ=255)`  
                      `ggATGCGGTAGGTCCGAATATAAGGATGGAGACTTTGACATTCTTGCGGTTGTGGTTGACGAAGATGTGCTTATTTTCACATGGGACGAAGTAAAAGGTAAGACATCCATGTGTGTCGGCAAGAGAAACAAAGGCATAAAACTATAGGAGAAATTATTATGGATATGACAAAGAAACTTAAAGTGTCCTTCGACGTTACCGCAAAGATGTCGTCTGACGTTCAGGCAATCTTAGAGAAAGATATGCTGCATCTATGTAAGCAGGTCAGCTCAGGTGCGCTTGTCCCCAATGGTAAACAGaa                                                                                                                                                                                                                                                                                       >  1:97687/1‑301 (MQ=255)`  
                       `gATGCGGTAGGTCCGAATATAAGGATGGAGACTTTGACATTCTTGCGGTTGTGGTTGACGAAGATGTGCTTATTTTCACATGGGACGAAGTAAAAGGTAAGACATCCATGTGTGTCGGCAAGAGAAACAAAGGCATAAAACTATAGGAGAAATTATTATGGCTATGACAAAGAAATTTAAAGTGTCCTTCGACGTTACCGCCAAGATGTCGTCTGACGTTCAGGCAATCTTAGAGAAAGATATGCTGCATCTATGTAAGCAGGTCGGCTCAGGTGCGATTGTCCCCAATGGTAAACAGAAg                                                                                                                                                                                                                                                                                      >  1:14145/1‑301 (MQ=255)`  
                           `cGGTAGGTCCGAATATAAGGATGGAGACTTTGACATTCTTGCGGTTGTGGTTGACGAAGATGTGCTTATTTTCACATGGGACGAAGTAAAAGGTAAGACATCCATGTGTGTCGGCAAGAGAAACAAAGGCATAAAACTATAGGAGAAATTATTATGGCTATGACAAAGAAATTTAAAGTGTCCTTCGACGTTACCGCAAAGATGTCGTCTGACGTTCAGGCAATCTTAGAGAAAGATATGCTGCATCTATGTAAGCAGGTCGGCTCAGGTGCGATTGTCCCCAATGGTAACCAGAAGGaaa                                                                                                                                                                                                                                                                                  >  1:21104/1‑301 (MQ=255)`  
                               `aGGTCCGAATATAAGGATGGAGACTTTGACATTCTTGAGGTTGTGGTTGACGAAGATGTGCTTATTTTCACATGGGACGAAGTAAAAGGTAAGACATCCATGTGTGTCGGCAAGAGAAACAAAGGCATAAAACTATAGGAGAAATTATTATGGCTATGACAAAGAAATTTAAAGTGTCCTTCGACGTTACCGCAAAGATGTCGTCTGACGTTCAGGCAATCTTAGAGAAAGATATGCTGCATCTATGTAAGCAGGTCGGCTAAGGTGCGATTGTCCCCAATGGTAAACAGAAGGAAATGAt                                                                                                                                                                                                                                                                              <  1:58043/301‑1 (MQ=255)`  
                                 `gTCCGAATATAAGGATGGAGACTTTGACATTCTTGCGGTTGTGGTTGACGAAGATGTGCTTATTTTCACATGGGACGAAGTAAAAGGTAAGACATCCATGTGTGTCGGCAAGAGAAACAAAGGCATAAAACTATAGGAGAAATTATTATGGCTATGACAAAGAAATTTAAAGTGTCCTTCGACGTTACCGCAAAGATGTCGTCTGACGTTCAGTCAATCTTAGAGAAAGATATGCTGCATCTATGTAAGCAGGTCGGCTCATGTTCGATTGTCCCCAATggctgtctcttatacacatctc                                                                                                                                                                                                                                                                            >  1:282030/1‑281 (MQ=255)`  
                                       `atatAAGGATGGAGACTTTGACATTCTTGCGGTTGTGGTTGACGAAGATGTGCTTATTTTCACATGGGACGAAGTAAAAGGTAAGACATCCATGTGTGTCGGCAAGAGAAACAAAGGCATAAAACTATAGGAGCAATTATTATGGCTATGACAAAGAACTTTAAAGTGTCCTTCTACGTTACCGCAAAGATGTCGTCTGACGTTCAGGCCATCTTAGAGAAAGACATACTGCATCTATGTAACCAGGTCTGCTCAGTTGCTATTGTCCCCACTGGTAAATCGAAGGATATGATTGTCCAGt                                                                                                                                                                                                                                                                      >  1:335675/1‑301 (MQ=255)`  
                                       `atatAAGGATGGAGACTTTGACATTCTTGCGGTTGTGGTTGACGAAGATGTGCTTATTTTCACATGGGACGAAGTAAAAGGTAAGACATCCATGTGTGTCGGCAAGAGAAACAAAGGCATAAAACTATAGGAGAAATTATTATGGCTATGACAAAGAAATTTTAAGTGTCCTTCGACGTTACCGCAAAGATGTCGTCTGACGTTCAGGCAATCTTAGAGAAAGATATGCTGCATCTATGTAAGCAGGTCGGCTCAGGTGCGATTGTCCCCCATGGtctggctcttatacacatctccgagc                                                                                                                                                                                                                                                                      >  1:193154/1‑276 (MQ=255)`  
                                       `atatAAGGATGGAGACTTTGACATTCTTGCGGTTGTGGTTGACGAAGATGTGCTTATTTTCACATGGGACGAAGTAAAAGGTAAGACATCCATGTGTGTCGGCAAGAGAAACAAAGGCATAAAACTATAGGAGAAATTATTATGGCTATGACAAAGAAATTTAAAGTGTCCTTCGACGTTACCGCAAAGATGTCGTCTGACGTTCAGGCAATCTTAGAGAAAGATATGCTGCATCTATGTAAGCAGGTCGGCTCAGGTGCGATTGTCCCCAATGGTAAACAGAAGGAAATGATTGTCCAGt                                                                                                                                                                                                                                                                      >  1:170119/1‑301 (MQ=255)`  
                                       `atatAAGGATGGAGACTTTGACATTCTTGCGGTTGTGGTTGACGAAGATGTGCTTATTTTCACATGGGACGAAGTAAAAGGTAAGACATCCATGTGTGTCGGCAAGAGAAACAAAGGCATAAAACTATAGGAGAAATTATTATGGCTATGACAAAGAAATTTAAAGTGTCCTTCGACGTTACCGCAAAGATGTCGTCTGACGTTCAGGCAATCTTAGAGAAAGATATGCTGCATCTATGTAAGCAGGTCGGCTCAGGTGCGATTGTCCCCAATGGTAAACAGAAGGAAATGATTGTCCAGt                                                                                                                                                                                                                                                                      >  1:303102/1‑301 (MQ=255)`  
                                        `tataAGGATGGAGACTTTGACATTCTTGCGGTTGTGGTTGACGAAGATGTGCTTATTTTCACATGGGACGAAGTAACAGGTAAGACATCCATGTGTGTCGGCAAGAGAAACAAAGTCATAAAACTATAGGAGAAATTATTATGGCTATGACAAAGAAATTTAAAGTGTCCTTCGACGTTACCGCAAAGATGTCGTCTGACGTTCAGGCAATCTTAGAGAAAGATATGCTTCATCTATTTAAGCAGTTCGGCTCAGGTGCGATTGTCCCCAATGGTAAACAGAAGGAAATGATTGTCCAGtt                                                                                                                                                                                                                                                                     >  1:151056/1‑301 (MQ=255)`  
                                              `taTGGAGACTTTGACATTCTTGCGGTTGTGGTTGACGAAGATTTGCATATTTTCACATGGGACGAAGTAAAAGGTAATACATCCATTTGTGTCGGCAAGAGAAACAAAGGCATAAAACTATAGGAGAGATTATTATGGCTATGACAAAGAAATTTAAAGTGTCCTTCGACGTTACCGCAAAGATGTCGTCTGACGTTCAGGCAATCTTAGAGAAAGAGATGCTGCATCTATGTAAGCAGGTCGGCTCAGGTGCGATTGTCCCCAATGGTAAACAGAAGGAAATGATTGTCCAGTTCCTGac                                                                                                                                                                                                                                                               <  1:173461/300‑1 (MQ=255)`  
                                              `gATGGAGACTTTGACATTCTTGCGGTTGTGGTTGACGAAGATGTGCTTATTTTCACATGGGACGAAGTAAAAGGTAAGACATCCATGTGTGTCGGCAAGAGAAACAAAGGCATAAAACTATAGGAGAAATTATTATGGCTATGACAAAGAAATTTAAAGTGTCCTTCGACGTTACCGCAAAGATGTCGTCTGACGTTCAGGCAATCTTAGAGAAAGATATGCTGCATCTATGTAAGCAGGTCGGCTCAGGTGCGATTTTCCCCAATGGTAAACAGAAGGAAATGATTGTCCAGTTCCTGac                                                                                                                                                                                                                                                               >  1:133506/1‑301 (MQ=255)`  
                                                      `cTTTGACATTCTTGCGGTTGTGGTTGACGAAGATGTGCTTATTTTCACATGGGACGAAGTAAAAGGTAAGACATCCATGTGTGTCGGCAAGAGAAACAAAGGCATAAAACTATAGGAGAAATTATTATGGCTATGACAAAGAAATTTAAAGTGTCCTTCGACGTTACCGCAAAGATGTCGTCTGACGTTCAGGCAATCTTAGAGAAAGATATGCTGCATCTATGTAAGCAGGTCGGCTCAGGTGCGATTGTCCCCAATGGTAAACAGAAGGAAATGATTGTCCAGTTCCTGACACACGGTa                                                                                                                                                                                                                                                       >  1:278549/1‑301 (MQ=255)`  
                                                      `atttGACATGCTTGGGTTTGTTGTTGACGAAGATGTGCTAATTTTCACATGGGAAGAAGTAAAAGGAAAGACAAACATGTGTGTCGGCAAGAGAAACAAAGGCATAAAACTATAGGAGAAATTATTATGGCTATGACAAAGAAATTTAAAGTGTCCTTCGACGTTACCGCAAAGATGTCGTCTGACGTTCAGGCAATCTTAGAGAAAGATATGCTGCATCTATGTAAGCAGGTCGGCTCAGGTGCGATTGTCCCCAATGGTAAACAGAAGGAAATGATTGACCAGTTCCTGACACACGGTa                                                                                                                                                                                                                                                       <  1:151948/300‑1 (MQ=255)`  
                                                          `gACATTCTTGCGGTTGTGGTTTACGTAGATGTGCTTATTTTCATATGGGACGAAGTAAAAGGTAAGACATCCATGTGTGTCGGCCAGTGAAACAAAGGCATAAAACTATAGGAGATATTATTATTGCTATTCCAAAGAAATTTAAAGTGTCCTTCTACGTTACCGCAAATATGTCTTCTGACGTTCAGGCAATCTTAGAGAAAGATATGCTGCATCTATGTAAGCAGGTCGGCTCAGGTGCGATTGTCCCCAATGGTAAACAGAAGGAAATGATTGTCCAGTTCCGGACACACGGTATGGa                                                                                                                                                                                                                                                   <  1:93995/301‑1 (MQ=255)`  
                                                          `gACATTCTTGCGGTTGTGGTTGACGAAGATGTGCTTATTTTCACATGGGACGAAGTAAAAGGTAAGACATCCATGTGTGTCGGCAAGAGAAACAAAGGCATAAAACTATAGGAGAAATTATTATGGCTATGACAAAGAAATTTAAAGTGTCCTTCGACGTTACCGCAAAGATGTCGTCTGACGTTCAGGCAATCTTAGAGAAAGATATGCTGCATCTATGTAAGCAGGTCGGCTCAGGTGCGATTGTCCCCAATGGTAAACAGAAGGGAATGATTGTCCAGTTCCTGACACACGGTATGGa                                                                                                                                                                                                                                                   <  1:51517/301‑1 (MQ=255)`  
                                                          `gACATTCTTGCGGTTGTGGTTGACGAAGATGTGCTTATTTTCACATGGGACGAAGTAAAAGGTAAGACATCCATGTGTGTCGGCAAGAGAAACAAAGGCATAAAACTATAGGAGAAATTATTATGGCTATGACAAAGAAATTTAAAGTGTCCTTCGACGTTACCGCAAAGATGTCGTCTGACGTTCAGGCAATCTTAGAGAAAGATATGCTGCATCTATGTAAGCAGGTCGGCTCAGGTGCGATTGTCCCCAATGGTAAACAGAAGGAAATGATTGTCCAGTTCCTGACACACGGTATGGa                                                                                                                                                                                                                                                   <  1:122349/301‑1 (MQ=255)`  
                                                          `gACATTCTTGCGGTTGGGAATGACGAAGATGAGCTTATTTTCACATGGGACGAAGTAAAAGGTAAGACATCCATGGGTGTCTGCAAGAGAAACAAAGGCATAAAACTATAGGAGAAATTATTATGACTATGACAAAGAAATATAAAGTGTCCTTCGACGTTACCGCAAAGATGTCGTCTGACGTTCAGGCAATCTTAGAGAAAGATATGCTGCATCTATGTAAGCAGGTCGGCTCAGGTGCGATTGTCCCCAATGGTAAACAGAAGGAAATGATTGTCCAGTTCCTGACACACGGTATGGa                                                                                                                                                                                                                                                   <  1:81368/301‑1 (MQ=255)`  
                                                           `aCATTCTTGCGGTTGTGGTTGACGAAGATGTGCTTATTTTCACATGGGACGAAGTAAAAGGTAAGACATCCATGTGTGTCGGCAAGAGAAACAAAGGCATAAAACTATAGGAGAAATTATTATGGCTATGACAAAGAAATTTAAAGTGTCCTTCGACGTTACCGCAAAGATGTCGTCTGACGTTCAGGCAATCTTAGAGAAAGATATGCTGCATCTATGTAAGCAGGTCGGCTCAGGTGCGATTGTCCCCAATGGTAAACAGAAGGAAATGATTGTCCAGTTCCTGACACACGGTATGGaa                                                                                                                                                                                                                                                  <  1:13140/301‑1 (MQ=255)`  
                                                            `taTTCTTGCGGTTGTGGTTGACGAAGATGTGCTTATTTTCACATGGGACGAAGTAAAAGGTAAGACATCCATGTGTGTCGGCAAGAGAAACAAAGGCATAAAACTATAGGAGAAATTATTATGGCTATGACAAAGAAATTTAAAGTGTCCTTCGACGTTACCGCAAAGACGTCGTCTGACGTTCAGGCAATCTTAGAGAAAGATATGCTGCATCTATGTAAGCAGGTCGGCTCAGGTGCGATTGTCCCCAATGGTAAACAGAAGGAAATGATTGTCCAGTTCCTGACACACGGTATGGAAg                                                                                                                                                                                                                                                 <  1:107994/300‑1 (MQ=255)`  
                                                            `cATACTTGCGGTTGTGGTTGACGAAGATGTGCTCATTTTCACATGGGACGAAGTAAAATGTAAGACATCCATGTGTGTCGGCAAGAGAAACAAAGGCATAAAACTATAGGAGAAATTATTATGGCTATGACAAAGAAATTTAAAGTGTCCTTCAACGTTACCGCAAAGATGTCGTCTGACGTTCAGGCAATCTTAGAGAAAGATATGCTGCATCTATGTAAGCAGGTCGGCTCAGGTGCGATTGTCCCCAATGGTAAACAGAAGGAAATGATTGTCCAGTTCCTGACACACGGTATGGAAg                                                                                                                                                                                                                                                 <  1:315085/301‑1 (MQ=255)`  
                                                            `aaTTCTTGCGGTTGTGGTTGACGAAGATGTGCTTATTTTCACATGGGACGAAGTAAAAGGTAAGACATCCATGTGTGTCGGCAAGAGAAACAAAGACATAAAACTATAGGAGAAATTATTATGGCTATGACAAAGAAATTTAAAGTGTCCTTCGACGTTACCGCAAAGATGTCGTCTGACGTTCAGGCAATCTTAGAGAAAGATATGCTGCATCTATGTAAGCAGGTCGGCTCAGGTGCGATTGTCCCCAATGGTAAACAGAAGGAAATGATTGTCCAGTTCCTGACACACGGTATGGAAg                                                                                                                                                                                                                                                 <  1:91370/300‑1 (MQ=255)`  
                                                            `aaTTCTTGCGGTTGTGGTTGACGAAGAGGTGCTTATTTTCACATGGGACGAAGTAAAAGGTAAGACATCCATGTGTGTCGGCAAGAGAAACAAAGGCATAAAACTATAGGAGAAATTATTATGGCTATGACAAAGAAATTTAAAGTGTCCTTCGACGTTACCGCAAAGATGTCGTCTGACGTTCAGGCAATCTTAGAGAAAGATATGCTGCATCTATGTAAGCAGGTCGGCTCAGGTGCGATTGTCCCCAATGGTAAACAGAAGGAAATGATTGTCCAGTTCCTAACACACGGTATGGAAg                                                                                                                                                                                                                                                 <  1:276541/300‑1 (MQ=255)`  
                                                            `aaTTCGTGCGGTTGTGGTTGACGAAGATGTGCTTATTTTCACATGGGACGAAGTAAAAGGTAAGACATCCATGTGTGTCGGCAAGAGAAACAAAGGCATAAAACTATAGGAGAAATTATTATGGCTATGACAAAGAAATTTAAAGTGTCCTTCGACGTTACCGCAAAGATGTCGTCTGACGTTCAGGCAATCTTAGAGAAAGATATGCTGCATCTATGTAAGCAGGTCGGCTCAGGTGCGATTGTCCCCAATGGTAAACAGAAGGAAATGATTGTCCAGTTCCTGACACACGGTATGGAAg                                                                                                                                                                                                                                                 <  1:220184/300‑1 (MQ=255)`  
                                                               `tCTTGTGGTTGTGGTTGACGAAGATGTGCTTATTTTCACATGGGACGAAGTAAAAGGTAAGACATCCATGTGTGTCGGCAAGAGAAACAAAGGCATAAAACTATAGGAGAAATTATTATGGCTATGACAAAGAAATTTAAAGTGTCCTTCGACGTTACCGCAAAGATGTCGTCTGACGTTCAGGCAATCTTAGAGAAAGATATGCTGCATCTATGTAAGCAGGTCGGCTCAGGTGCGATTGTCCCCAATGGTAAACAGAAGGAAATGATTGTCCAGTTCCTGACACACGGTATGGAAGGAt                                                                                                                                                                                                                                              >  1:160905/1‑301 (MQ=255)`  
                                                                    `cGGTTGTGGTTGACGAAGATGTGCTTATTTTCACATGGGACGAAGCAAAAGGTAAGACATCCATGTGTGTCGGCAAGAGAAACAAAGGCATAAAACTATAGGAGAAATTATTATGGCTATGACAAAGAAATTTAAAGTGTCCTTCGACGTTACCGCAAAGATGTCGTCTGACGTTCAGGCAATCTTTGAGAAAGATATGCTGCATTTATGTAAGCAGGTCGGCTCAGGTGCGATTGTATCTAATTGTAAACAGAAGTAAATGATTGTCTAGTTCCTGACACTCGGTATGGTAGGAttgttg                                                                                                                                                                                                                                         >  1:331758/1‑301 (MQ=255)`  
                                                                         `gtgGTTGACGAAGATGTGCTTATTTTCACATGGGACGAAGTAAAAGGTAAGACATCCATGTGTGTCGGCAAGAGAAACAAAGGCATAAAACTATAGGAGAAATTATTATGGCTATGACAAAGAAATTTAAAGTGTCCTTCGACGTTACCGCAAAGATGTCGTCTGACGTTCAGGCAATCTTAGAGAAAGATATGCTGCATCTATGTAAGCAGGTCGGCTCAGGTGCGATTGTCCCCAATGGTAAACAGAAGGAAATGATTGTCCAGTTCCTGACACACGGTATGGAAGGATTGATGACAtt                                                                                                                                                                                                                                    >  1:23671/1‑301 (MQ=255)`  
                                                                              `tGACGAAGATGTGCTTATTTTCACATGGGACGAAGTAAAAGGTAAGACATCCATGTGTGTCGGCAAGAGAAACAAAGGCATAAAACTATAGGAGAAATTATTATGGCTATGACAAAGAAATTTAAAGTGTCCTTCGACGTTACCGCAAAGATGTCGTCTGACGTTCAGGCAATCTTAGAGAAAGATATGCTGCATCTATGTAAGCAGGTCGGCTCAGGTGCGATTGTCCCCAATGGTAAACAGAAGGAAATGATTGTCCAGTTCCTGACACACGGTATGGGAGGATCGATGACATTCgtag                                                                                                                                                                                                                               <  1:4644/301‑1 (MQ=255)`  
                                                                              `aaagagacaggtgCTTATTTTCACATGGGACGAAGTAAAAGGTAAGACATCCATGTGTGTCGGCAAGAGAAACAAAGGCATAAAACTATAGGAGAATTTATTATGGCTATGACAAAGAAATTTAAAGTGTCCTTCGACGTTACCGCAAAGATGTCGTCTGACGTTCAGGCAATCTTAGAGAAAGATATGCTGCATCTATGTAAGCAGGTCGGCTCAGGTGCGATTGTCCCCAATGGTAAACAGAAGGAAATGATTGTCCAGTTCCTGACACACGGTATGGAAGGATTGATGACATTCgtag                                                                                                                                                                                                                               <  1:197226/291‑1 (MQ=255)`  
                                                                                `aCGAAGATGTGCTTATTTTCACTTGGGACGAAGTAAAAGGTAAGACATCCATGTGTGTCGGCAAGAGAAACAAAGGCATAAAACTATAGGAGAAATTATTATGGCTATGACAAAGAAATTTAAAGTGTCCTTCGACGTTACCGCAAAGATGTCGTCTGACGTTCAGGCAATCTTAGAGAAAGATATGCTGCATCTATGTAAGCAGGTCGGCTCAGGTGCGATTGTCCCCAATGGTAAACAGAAGGAAATGATTGTCCAGTTCCTGACACACGGTATGGAAGGATTGATGACATTCgtagta                                                                                                                                                                                                                             >  1:222735/1‑301 (MQ=255)`  
                                                                                `aCGAAGATGTGCTTATTTTCACATGGGACGAAGTAAAAGGTAAGACATCCATGTGTGTCGGCAAGAGAAACAAAGGCATAAAACTATAGGAGAAATTATTATGGCTATGACAAAGAAATTTAAAGTGTCCTTCGACGTTACCGCAAAGATGTCGTCTGACGTTCAGGCAATCTTAGAGAAAGATATGCTGCATCTATGTAAGCAGGTCGGCTCAGGTGCGATTGTCCCCAATGGTAAACAGAAGGAAATGATTGTCCAGTTCCTGACACACGGTATGGAAGGATTGATGACATTCgtagta                                                                                                                                                                                                                             <  1:138116/301‑1 (MQ=255)`  
                                                                                   `aaGATGTGCTTATTTTCAAATGGGACGAAGTAAAAGGTAAGACCTCCATGTGTGTCGGCAAGAGAAACAAAGGCATAAAACTATAGGAGAAATTATTATGGCTATGACAAAGAAATTTAAATTGTCCTTCGACGTTACCGCAAAGATGTCGTCTGACGTTCAGGCAATCTTAGAGAAAGATATGCTGCATCTATGTAAGCAGGTCGGCTCAGGTGCGATTGTCCCCAATGGTAAACAGAAGGAAATGATTGTCCAGTTCCTGACACACGGTATGGAAGGATTGATGACATTCGTAgtacgt                                                                                                                                                                                                                          <  1:7909/301‑1 (MQ=255)`  
                                                                                        `gtgCTTATTTTCACATGGGACGAAGTAAAAGGTAAGACATCCATGTGTGTCGGCAAGAGAAACAAAGGCATAAAACTATAGGAGAAATTATTATGGCTATGACAAAGAAATTTAAAGTGTCCTTCGACGTTACCGCAAAGATGTCGTCTGACGTTCAGGCAATCTTAGAGAAAGATATGCTGCATCTATGTAAGCAGGTCGGCTCAGGTGCGATTGTCCCCAATGGTAAACAGAAGGAAATGATTGTCCAGTTCCTGACACACGGTATGGAAGGATTGATGACATTCGTAGTACGTAcatc                                                                                                                                                                                                                     >  1:15594/1‑301 (MQ=255)`  
                                                                                        `atgcgtgtgttCAAATGGGGCGAAAGAAAAGGTAAGGCATCCATGTGTGTCGGAAAGGGAAACAAAGTCATAAAACGTTAGGAGAAATTATTATTGCTATGAAGAAGAAATTTAAAGTGTCCTTCGACGTTACCGCAAAGATGTCGTTTGACGTTCAGGCAATCTTAGAGAAAGATATGCTGAATGTATGTAAGCAGGTCGGCTCAGGTGCGATTGTCCCCAATGGTAAACAGAAGGAAATGATTGTCCAGTTCCTGACACACGGTATGGAAGGATTGATGACATTCGTAGTACGTAcatc                                                                                                                                                                                                                     <  1:202028/292‑1 (MQ=255)`  
                                                                                             `tATTTTCACATGGGACGAAGTAAAAGGTAAGACATCCATGTGTGTCGGCAAGAGAAACAAAGGCACAAAACTATAGGAGAAATTATTATGGCTATGACAAAGAAATTTAAAGTGTCCTTCGACGTTACCGCAAAGATGTCGTCTGACGTTCAGGCAATCTTAGAGAAAGATATGCTGCATCTATGTAAGCAGTTCGGCTCAGGTGCGATTGTCCCCAATGGTAAACAGAAGGAAATGATTGTCCAGTTCCTGACACACGGTATGGAAGGATTGAGGACATTCGTAGTACGTAAATCAtttt                                                                                                                                                                                                                >  1:26326/1‑300 (MQ=255)`  
                                                                                              `ataagagacagggACGAAGTAAAAGGTATGACATCCATGTGTGTCGGCAAGAGAAACAAAGGCATAAAACTATAGGAGAAATTATTATGGCTATGACAAAGAAATTTAAAGTGTCCTTCGACGTTACCGCAAAGATGTCGTCTGACGTTCAGGCAATCTTAGAGAAAGATATGCTGCATCTATGTAAGCAGGTCGGCTCAGGTGCGATTGTCCCCAATGGTAAACAGAAGGAAATGATTGTCCAGTTCCTGACACACGGTATGGAAGGATTGATGACATTCGTAGTACGTACATCATTTCg                                                                                                                                                                                                               <  1:29575/291‑1 (MQ=255)`  
                                                                                                         `ggACGAAGTAAAAGGTAAGACATCCATGTGTGTCGGCAAGAGAAACAAAGGCATAAAACTATAGGAGAAATTATTATGGCTATGACAAAGAAATTTAAAGTGTCCTTCGACGTTACCGCAAAGATGTCGTCTGACGTTCAGGCAATCTTAGAGAAAGATATGCTGCATCTATGTAAGCAGGTCGGCTCAGGTGCGATTGTCCCCAATGGTAAACAGAAGGAAATGATTGTCCAGTTCCTGACACACGGTATGGAAGGATTGATGACATTCGTAGTACGTACATCATTTCGTGAGGCCATTa                                                                                                                                                                                                    >  1:77166/1‑301 (MQ=255)`  
                                                                                                         `ggACGAAGTAAAAGGTAAGACATCCATGTGTGTCGGCAAGAGAAACAAAGGCATAAAACTATAGGAGAAATTATTATGGCTATGACAAAGAAATTTAAAGTGTCCTTCGACGTTACCGCAAAGATGTCGTCTGACGTTCAGGCAATCTTAGAGAAAGATATGCTGCATCTATGTAAGCAGGTCGGCTCAGGTGCGATTGTCCCCAATGGTAAACAGAAGGAAATGATTGTCCAGTTCCTGACACACGGTATGGAAGGATTGATGACATTCGTAGTACGTACATCATTTCGTGAGGCCATTa                                                                                                                                                                                                    >  1:16577/1‑301 (MQ=255)`  
                                                                                                            `cGAAGTAAAAGGTAAGACATCCAAGTGTGCCGGCAAGAGAAACAAAGGCATAAAACTATAGGAGAAATTATTATGGCTATGACAAAGAAATTTAAAGTGTCCTTCGACGTTACCGCAAAGATGTCGTCTGACGTTCAGGCAATCTTAGAGAAAGATATGCTGCATCTATGTAAGCAGGTCGGCTCAGGTGCGATTGTCCCCAATGGTAAACAGAAGGAAATGATTGTCCAGTTCCTGACACACGGTATGGAAGGATTGATGACATTCGTAGTACGTACATCATTTCGTGAGGCCATTAAgg                                                                                                                                                                                                 <  1:293337/301‑1 (MQ=255)`  
                                                                                                             `aaaGTAAAAGGTAATACATCCATGTGTGTCGGCAAGAGAAACAAAGGCATAAAACTATAGGAGAAATTATTATGGCTATGAAAAAGAAATTTAAAGTGTCCTTCGACGTTACCGCAAAGAGGTCGTCTGACGTTCAGGCAATCTTAGAGAAAGATATGCTGCATCTATGTAAGCAGGTCGGCTCAGGTGCGATTGTCCCCAATGGTAAACAGAAGGAAATGATTGTCCAGTTCCTGACACACGGTATGGAAGGATTGATGACATTCGTAGTACGTACATCATTTCGTGAGGCCATTAAGGa                                                                                                                                                                                                <  1:209136/300‑1 (MQ=255)`  
                                                                                                              `aaGTAAAAGGGAAGACATACATGGGTGTCGGCAAGAGAAACAAAGGCATAAAACTATAGGAGAAATTATTATGGCTATGAAAAAGAAATTTAAAGTGTCCTTCGACGTTACCGCAAAGATGTCGTCTGACGTTCAGGCAATCTTAGAGAAAGATATGCTGCATCTATGTAAGCAGGTCGGCTCAGGTGCGATTGTCCCCAATGGTAAACAGAAGGAAATGATTGTCCAGTTCCTGACACACGGTATGGAAGGATTGATGACATTCGTAGTACGTACATCATTTCGTGAGGCCATTAAGGAc                                                                                                                                                                                               <  1:260111/301‑1 (MQ=255)`  
                                                                                                               `aGTAAAAGGTAAGACATCCATGTGTGTCGGCAAGAGAAACAAAGGCATAAAACTATAGGAGAAATTATTATGGCTATGACAAAGAAATTTAAAGTATCCTTCGACGTTACCGCAAAGATGTCGTCTGACGTTCAGGCAATCTTAGAGAAAGATATGCTGCATCTATGTAAGCAGGTCGGCTCAGGTGCGATTGTCCCCAATGGTAAACAGAAGGAAATGATTGTCCAGTTCCTGACGCACGGTATGGAAGGATTGATGACATTCGTAGTACGTACATCATTTAGTGAGGCCATTAAGGACa                                                                                                                                                                                              >  1:299830/1‑301 (MQ=255)`  
                                                                                                                `gTAAAAGGTAAGACATCCATGTGTGTCGGCAAGAGAAACAAAGGCATAAAACTATAGGAGAAATTATTATGGCTATGACAAAGAAATTTAAAGTGTCCTTCGACGTTACCGCAAAGATGTCGTCTGACGTTCAGGCAATCTTAGAGAAAGATATGCTGCATCTATGTAAGCAGGTCGGCTCAGGTGCGATTGTCCCCAATGGTAAACAGAAGGAAATGATTGTCCAGTTCCTGACACACGGTATGGAAGGATTGATGACATTCGTAGTCCGTACATCATTTAGTgctgtcgcttatacaca                                                                                                                                                                                             >  1:176526/1‑285 (MQ=255)`  
                                                                                                                          `acagaTCCATGTGTGTAGGCAAGAGAAACAAAGGCATAAAACTATAGGAGAAATTATTATGGCTATGACAAAGAAATTTAAAGTGTCCTTCGACGTTACCGCAAAGATGTCGTCTGACGTTCAGGCAATCTTAGAGAAAGATATGCTGCATCTATGTAAGCAGGTCGGCTCAGGTGCGATTGTCCCCAATGGTAAACAGAAGGAAATGATTGTCCAGTTCCTGACACACGGTATGGAAGGATTGATGACATTCGTAGTACGTACATCATTTCGTGAGGCCATTAAGGACATGCACGAagag                                                                                                                                                                                   <  1:267811/297‑1 (MQ=255)`  
                                                                                                                          `aGACATCCATGTGTGGCGGCAAGAGAAACAAAGGCATAAAACTATAGGAGAAATTATTATGGCTATGACAAAGAAATTTAAAGTGTCCTTCGACGTTACCGCAAAGATGTCGTCTGACGTTCAGGCAATCTTAGAGAAAGATATGCTGCATCTATGTAAGCAGGTCGGCTCAGGTGCGATTGTCCCCAATGGTAAACAGAAGGAAATGATTGTCCAGTTCCTGACACACGGTATGGAAGGATTGATGACATTCGTAGTACGTACATCATTTCGTGAGGCCATTAAGGACATGCACGAagag                                                                                                                                                                                   <  1:250043/301‑1 (MQ=255)`  
                                                                                                                            `aCATCCATGTGTGTCGGCAAGAGAAACAAAGGCATAAAACTATAGGAGAAATTATTATGGCTATGACAAAGAAATTTAAATTGTCCTACGACGTTACCGCAAAGATGTCGTCTGACGTTCAGGCAATCTTAGAGAAAGATATGCTGAATCTATGTAAGCAGGTCGGCTCAGGTGCGATTGTCCCCAATGGTAAACAGAAGGAAATGATTGTCCAGTTCCTGACACACGGGATGGAAGGATTGATGACATTCGTAGTACGTACATCATTTCGTGAGGCCATTAAGGACATGCACGAAGAGTa                                                                                                                                                                                 <  1:181763/301‑1 (MQ=255)`  
                                                                                                                             `gaTCCATGTGTGTCGGCAAGAGAAACAAAGGCATAAAACTATAGGAGAAATTATTATGTCTATGACAAAGAAATTTAAAGTGTCCTTCGACGTTACCGCAAAGATGTCGTCTGACGTTCAGGCAATCTTAGAGAAAGATATGCTGCATCTATGTAAGCAGGTCGGCTCAGGTGCGATTGTCCCCAATGGTAAACAGAAGGAAATGATTGTCCAGTTCCTGACACACGGTATGGAAGGATTGATGACATTCGTAGTACGTACATCATTTCGTGAGGCCATTAAGGACATGCACGAAGAGTAt                                                                                                                                                                                >  1:6717/2‑301 (MQ=255)`  
                                                                                                                             `cATCCATGTGTGTCGGCAAGAGAAACAAAGGCATAAAACTATAGGAGAAATTATTATGGCTATGACAAAGAAATTTAAAGTGTCCTTCGACGTTACCGCAAAGATGTCGTCTGACGTTCAGGCAATCTTAGAGAAAGATATGCTGCATCTATGTAAGCAGGTCGGCTCAGGTGCGATTGTCCCCAATGGTAAACAGAAGGAAATGATTGTCCAGTTCCTGACACACGGTATGGAAGGATTGATGACATTCGTAGTACGTACATCATTTCGTGAGGCCATTAAGGACATGCACGAAGAGTAt                                                                                                                                                                                >  1:206135/1‑301 (MQ=255)`  
                                                                                                                              `aTCCATGTGTGTCGGCAAGAGAAACAAAGGCATAAAACTATAGGAGAAATTATTATGGCTATGACAAAGAAATTTAAAGTGTCCTTCGACGTTACCGCAAAGATGTCGTCTGACGTTCAGGCAATCTTAGAGAAAGATATGCTGCATCTATGTAAGCAGGTCGGCTCAGGTGCGATTGTCCCCAATGGTAAACAGAAGGAAATGATTGTCCAGTTCCTGACACACGGTATGGAAGGATTGATGACATTCGTAGTACGTACATCATTTCGTGAGGCCATTAAGGACATGCACGAAGAGTAta                                                                                                                                                                               >  1:327054/1‑300 (MQ=255)`  
                                                                                                                               `cccatggtggtCGGAAAAAGAAACAAAGGCATAAAACTATAGGAGAAATGATTATGGCTATGACAAAGAAATTTAAAGTGTCCTTCGACGTTACCGCAAAGATGTCGTCTGACGTTCAGGCAATCTTAGAGAAAGATATGCTGCATCTATGTAAGCAGGTCGGCTCAGGTGCGTTTGACCCCAATGGTAAACAGAAGGAAAGGATTGTCCAGTTCCTGACACACGGTATGGAAGGATTGATGACATTCGTAGTACGTACATCATTTCGTGAGGCCATTAAGGACATGCACGAAGAGTATGc                                                                                                                                                                              <  1:41916/292‑1 (MQ=255)`  
                                                                                                                                      `gtgtCGGCAAGAGAAACAAAGGCATAAAACTATAGGAGAAATTATTATGGCTATGACAAAGAAATTTAAAGTGTTCTTCGACGTTACCGCAAAGATGTCGTCTGACGTTCAGGCAATCTTAGAGAAAGATATGCTGCATCTATGTAAGCAGGTCGGCTCAGGTGCGATTGTCCCCAATGGTAAACAGAAGGAAATGATTGTCCAGTTCCTGACACACGGTATGGAAGGATTGATGACATTCGTAGTACGTACATCATTTCGTGATGCCATTAAGGACATGCACGAAGAGTATGCAGATAAg                                                                                                                                                                       >  1:186632/1‑301 (MQ=255)`  
                                                                                                                                      `gtgtCGGCAAGAGAAACAAAGGCATAAAACTATAGGAGAAATTATTATGGCTATGACAAAGAAATTTAAAGTGTCCTTCGACGTTACCGCAAAGATGTCGTCTGACGTTCAGGCAATCTTAGAGAAAGATATGCTGCATCTATGTAAGCAGGTCGGCTCAGGTGCGATTGTCCCCAATGGTAAACAGAAGGAAATGATTGTCAAGTTCCTGACACACGGTATGGAAGGATTGATGACATTCGTAGTACGTACATCATTTCGTGAGGCCATTAAGGACATGCACGAagcggtctcttataca                                                                                                                                                                       >  1:83526/1‑287 (MQ=255)`  
                                                                                                                                      `gtgtCGGCAAGAGAAACAAAGGCATAAAACTATAGGAGAAATTATTATGGCTATGACAAAGAAATTTAAAGTATCCTTCGACGTTACCGCAAAGATGTCGTCTGACGTTCAGGCAATCTTAGAGAAAGATATGCTGCATCTATGTAAGCAGGTCGGCTCAGGTGCGATTGTCCCCAATGGTAAACAGAAGGAAATGATTGTCCAGTTCCTGACACACGTTATGGCAGGATTGTTGACATTCGTAGTACGTACATCATTTCGTGAGGCCATTAAGGACATGCACGAAGAGTATGCAGATAAg                                                                                                                                                                       >  1:179331/1‑301 (MQ=255)`  
                                                                                                                                         `ctcggagatgtttataagagacagaaCTATAGGAGAAATTATTATGGCTATGACAAAGAAATTTAAAGTGTCCTTCGACGTTACCGCAAAGATGTCGTCTGACGTTCAGGCAATCTTAGAGAAAGATATGCTGCATCTATGTAAGCAGGTCGGCTCAGGTGCGATTGTCCCCAATGGTAAACAGAAGGAAATGATTGTCCAGTTCCTGACACACGGTATGGAAGGATTGATGACATTCGTAGTACGTACATCATTTCGTGAGGCCATTAAGGACATGCACGAAGAGTATGCAGATAAGGAc                                                                                                                                                                    <  1:36987/277‑1 (MQ=255)`  
                                                                                                                                                  `gaAACAAAGGCATAAAACTATAGGAGAAATGATTATGGCTATGACAAAGAAATTTAAAGTGTCCTTCGACGTTACCGCAAAGATGTCGTCTGACGTTCAGGCAATCTTAGAGAAAGATATGCTGCATCTATGTAAGCAGGTCGGCTCAGGTGCGATTGTCCCCAATGGTAAACAGAAGGAAATGATTGTCCAGTTCCTGACACACGGTATGGAAGGATTGATGACATTCGTAGTACGTACATCATTTCGTGAGGCCATTAAGGACATGCACGAAGAGTATGCAGATAAGGACTCTTTCaaa                                                                                                                                                           <  1:191410/301‑1 (MQ=255)`  
                                                                                                                                                   `aaaCAAAGGCATAAAACTATAGGAGAAATTATTATGGCTATGACAAAGAAATTTAAAGTGTCCTTCGACGTTACCGCAAAGATGTCGTCTGACGTTCAGGCAATCTTAGAGAAAGATATGCTGCATCTATGTAAGCAGGTCGGCTCAGGTGCGATTGTCCCCAATGGTAAACAGAAGGAAATGATTGTCCAGTTCCTGACACACGGTATGGAAGGATTGATGACATTCGTAGTACGTACATCCTTTCGTGAGGCCATTTAGGACATGCACGAAGAGTATGCAGATAAAGACTCTTTCaaaa                                                                                                                                                          >  1:228633/1‑300 (MQ=255)`  
                                                                                                                                                   `aaaCAAAGGAATAAAACGATAGGAGAAATTATTATGGCTATGACAAAGAAATTTAAAGTGTCATTCGACGTTACCGCAAAGATGTCGTCTGACGTTCAGGCAATCTTAGAGAAAGATATGCTGCATCTATGTAAGCAGGTCGGCTCAGGTGCGATTGTCCCCAATGGTAAACAGAAGGAAATGATTGTCCAGTTCCTGACACACGGTATGGAAGGATTGATGACATTCGTAGTACGTACATCATTTCGTGAGGCCATTAAGGACATGCACGAAGAGTATGCAGATAAGGACTCTTTCAAAc                                                                                                                                                          <  1:54807/301‑1 (MQ=255)`  
                                                                                                                                                            `cATAAAACTATAGGAGAAATTATTATGGCTATGACAAAGAAATTTAAAGTGTCCTTCGACGTTACCGCAAAGATGTCGTCTGACGTTCAGGCAATCTTAGAGAAAGATATGCTGCATCTATGTAAGCAGGTCGGCTCAGGTGCGATTGTCCCCAATGGTAAACAGAAGGAAATGATTGTCCAGTTCCTGACACACGGTATGGAAGGATTGATGACATTCGTAGTACGTACATCATTTCGTGAGGCCATTAAGGACATGCACGAAGAGTATGCAGATAAGGACTCTTTCAAACAATCTCCTg                                                                                                                                                 >  1:126278/1‑301 (MQ=255)`  
                                                                                                                                                                 `aaCTATAGGAGAAATTATTATGGCTATGACAAAGAAATTTAAAGTGTCCTTCGACGTTACCGCAAAGATGTCGTCTGACGTTCAGGCAATCTTAGAGAAAGATATGCTGCATCTATGTAAGCAGGTCGGCTCAGGTGCGATTGTCCCCAATGGTAAACAGAAGGAAATGATTGTCCAGTTCCTGACACACGGTATGGAAGGATTGATGACATTCGTAGTACGTACATCATTTCGTGAGGCCATTAAGGACATGCACGAAGAGTATGCAGATAAGGACTCTTTCAAACAATCTCCTGCAACa                                                                                                                                            >  1:342727/1‑301 (MQ=255)`  
                                                                                                                                                                 `aaCTATAGGAGAAATTATTATGGCTATGACAAAGAAATTTAAAGTGTCCTTCGACGTTACCGCAAAGATGTCGTCTGACGTTCAGGCAATCTTAGAGAAAGATATGCTGCATCTATGTAAGCAGGTCGGCTCAGGTGCGATTGTCCCCAATGGTAAACAGAAGGAAATGATTGTCCAGTTCCTGACACACGGTATGGAAGGATTGATGACATTCGTAGTACGTACATCATTTCGTGAGGCCATTAAGGACATGCACGAAGAGTATGCAGATAAGGACTCTTTCAAACAATCTCCTGCAACa                                                                                                                                            >  1:288797/1‑301 (MQ=255)`  
                                                                                                                                                                   `cTATAGGAGAAATTATTATGGCTATGACAAAGAAATTTAAAGTGTCCTTCGACGTTACCGCAAAGATGTCGTCTGACGTTCAGGCAATCTTAGAGAAAGATATGCTGCATCTATGTAAGCAGGTCGGCTCAGGTGCGATTGTCCCCAATGGTAAACAGAAGGAAATGATTGTCCAGTTCCTGACACACGGTATGGAAGGATTGATGACATTCGTAGTACGTACATCATTTCGTGAGGCCA‑TAAGGACATGAACGAAGAGTATGCAGATAAGGACTCTTTCAAACAATCCCCTGCAACAGTa                                                                                                                                         >  1:65290/1‑301 (MQ=255)`  
                                                                                                                                                                   `agataGGAGAAATTATTATGGCTATGACAAAGAAATTTAAAGTGTCCTTCGACGTTACCGCAAAGATGTCGGCTGACGTTCAGGCAAGCTTAGAGAAAGATATGCTGCATCTATGTAAGCAGGTCGGCTCAGGTGCGATTGTCCCCAATGGTAAACAGAAGGAAATGATTGTCCAGTTCCTGACACACGGTATGGAAGGATTGATGACATTCGTAGTACGTACATCATTTCGTGAGGCCATTAAGGACATGCACGAAGAGTATGCAGATAAGGACTCTTTCAAACAATCTCCTGCAACAGt                                                                                                                                          <  1:153985/299‑1 (MQ=255)`  
                                                                                                                                                                        `ggAGAAAATATGATGGCTATGACAAAGAAATTGAAAGTGTCCTTCGACGTTACCGCAAAGATGTCGTCTGACGTTCAGGCAATCTTAGAGAAAGATATGCTGCATCTAGGTAAGCAGGTCGGCTCAGGTGCGATTGTCCCCAATGGTAAACAGAAGGAAATGATTGTCCAGTTCCTGACACACGGTATGGAAGGATTGATGACATTCGTAGTACGTACATCATTTCGTGAGGCCATTAAGGACATGCACGAAGAGTATGCAGATAAGGACTCTTTCAAACAATCTCCTGCAACAGTACggg                                                                                                                                     <  1:52554/301‑1 (MQ=255)`  
                                                                                                                                                                           `gaAATGATTATGGCTATGACAAAGAAATTTAAAGTGTCCTTCGACGTTACCGCAAAGATGTCGTCTGACGTTCAGGCAATCTTAGAGAAAGATATGCTGCATCTATGTAAGCAGGTCGGCTCAGGTGCAATTGTCCCCAATGGTAAACAGAAGGAAATGATTGTCCAGTTCCTGACACACGGTATGGAAGGATTGATCACATTCGTAGTACGTACATCATTTCGTGAGGCCATTAAGGACATGCACGAAGAGTATGCAGATAAGGACTCTTTCAAACAATCTCCTGCAACAGTACGGGAgg                                                                                                                                  <  1:135382/301‑1 (MQ=255)`  
                                                                                                                                                                                      `ggCTATGACAAAGAAATTTAAAGTGTCCTTCGACGTTACCGCAAAGATGTCGTCTGACGTTCAGGCAATCTTAGAGAAAGATATGCTGCATCTATGTAAGCAGGTCGGCTCAGGTGCGATTGTCCCCAATGGTAAACAGAAGGAAATGATTGTCCAGTTCCTGACACACGGTATGGAAGGATTGATGACATTCGTAGTACGTACATCATTTCGTGAGGCCATTAAGGACATGCACGAAGAGTATGCAGATAAGGACTCTTTCAAACAATCTCCTGCAACAGTACGGGAGGAGTTCTGA‑TGt                                                                                                                      >  1:215171/1‑301 (MQ=255)`  
                                                                                                                                                                                          `aTGACAAAGAAATTTAAAGTGTCCTTCGACGTTACCGCAAAGATGTCGTCTGACGTTCAGGCAATCTTAGAGAAAGATATGCTGCATCTATGTAAGCAGGTCGGCTCAGGTGCGATTGTCCCCAATGGTAAACAGAAGGAAATGATTGTCCAGTTCCTGACACACGGTATGGAAGGATTGATGACATTCGTAGTACGTACATCATTTCGTGAGGCCATTAAGGACATGCACGAAGAGTATGCAGATAAGGACTCTTTCAAACAATCTCCTGCCACAGTACGGGAGGTGTTCTGA‑Tgctgtc                                                                                                                  >  1:257658/1‑296 (MQ=255)`  
                                                                                                                                                                                           `tGACAAAGAAATTTAAAGTGTCCTTCGACGTTACCGCAAAGATGTCGTCTGACGTTCAGGCAATCTTAGAGAAAGATATGCTGCATCTATGTAAGCAGGTCGGCTCAGGTGCGATTGTCCCCAATGGTAAACAGAAGGAAATGATTGTCCAGTTCCTGACACACGGTATGGAAGGATTGATGACATTCGTAGTACGTACATCATTTCGTGAGGCCATTAAGGACATGCACGAAGAGTATGCAGATAAGGACTCTTTCAAACAATCTCCTGCAACAGTACGGGCGGTGTTCTGACTGTCtct                                                                                                                  >  1:27206/1‑299 (MQ=255)`  
                                                                                                                                                                                           `tGACAAAGAAATTTAAAGTGTCCTTCGACGTTACCGCAAAGATGTCGTCTGACGTTCAGGCAATCTTAGAGAAAGATATGCTGCATCTATGTAAGCAGGTCGGCTCAGGTGCGATTGTCCCCAATGGTAAACAGAAGGAAATGATTGTCCAGTTCCTGACACACGGTATGGAAGGATTGATGACATTCGTAGTACGTACATCATTTCGTGAGGCCATTAAGGACATGCACGAAGAGTATGCAGATAAGGACTCTTTCAAACAATCTCCTGCAACAGTACGGGAGGTGTTCTGA‑TGTCTGAc                                                                                                                 >  1:63249/1‑301 (MQ=255)`  
                                                                                                                                                                                               `aggtgtataagaggcagccTTCGACGTTACCGCAAAGATGTCGTCTGACGTTCAGGCAATCTTAGAGAAAGATATGCGGCATCTATGTAAGCAGGTCGGCTCAGGTGCGATTGTCCCCAATGGTAAACAGAAGGAAATGATTGTCCAGTTCCTGACACACGGTATGGAAGGATTGATGACATTCGTAGTACGTACATCATTTCGTGAGGCCATTAAGGACATGCACGAAGAGTATGCAGATAAGGACTCTTTCAAACAATCTCCTGCAACAGTACGGGAGGTGTTCTGA‑TGTCTGACTAcc                                                                                                             <  1:320480/284‑1 (MQ=255)`  
                                                                                                                                                                                                    `aaTTTAAAGTGTCCTTCGACGTTACCGCAAAGATGTCGTCTGACGTTCAGGCAATCTTAGAGAAAGATATGCTGCATATATGTAAGCAGGTCGGCTCAGGTGCGATTGTCCCCAATGGTAAACAGAAGGAAATGATTATCCAGTTCCTGACACACGGTATGGAAGGATTGATGACATTCGTAGTACGTACATCATTTCGTGAGGCCATTAAGGACATGCACGAAGAGTATGCAGATAAGGACTCTTTCAAACAATCTCCTGCAACAGTACGGGAGGTGTTCTGA‑TGTCTGACTACCTGaaa                                                                                                        >  1:143634/1‑301 (MQ=255)`  
                                                                                                                                                                                                               `tCCTTCGACGTTACCGCAAAGATGTCGTCTGACGTTCAGGCAATCTTAGAGAAAGATATGCTGCATCTATGTAAGCAGGTCGGCTCAGGTGCGATTGTCCCCAATGGTAAACAGAAGGAAATGATTGTCCAGTTCCTGACACACGGTATGGAAGGATTGATGACATTCGTAGTACGTACATCATTTCGTGAGGCCATTAAGGACATGCACGAAGAGTATGCAGATAAGGACTCTTTCAAACAATCTCCTGCAACAGTACGGGAGGTGTTCTGA‑TGTCTGACTACCTGAAAGTGCTgcaact                                                                                             >  1:128110/1‑299 (MQ=255)`  
                                                                                                                                                                                                                         `ttACCGCAAAGATGTCGTCTGACGTTCAGGCAATCTTAGAGAAAGATATGCTGCATCTATGTAAGCAGGTCGGCTCAGGTGCGATTGTCCCCAATGGTAAACAGAAGGAAATGATTGTCCAGTTCCTGACACACGGTATGGAAGGATTGATGACATTCGTAGTACGTACATCATTTCGTGATGCCATTAAGGACATGCACGAATAGTATGCAGCTAAGGACTCTTTCAAACAATCTCCTGCAACAGTACGGGAGGTGTTCTGA‑TGTCTAACTACCTGAAAGTGCTGCAAGCAAGCAAAAgc                                                                                   >  1:189138/1‑300 (MQ=255)`  
                                                                                                                                                                                                                         `cagccGCAAAGATGTCGTCTGACGTTCAGGCAATCTTAGAGAAAGATATGCTGCATCTATGTAAGCAGGTCGGCTCAGGTGCGATTGTCCCCAATGGTAAACAGAAGGAAATGATTGTCCAGTTCCTGACACACGGTATGGAAGGATTGATGACATTCGTAGTACGTACATCATTTCGTGAGGCCATTAAGGACATGCACGAAGAGTATGCAGATAAGGACTCTTTCAAACAATCTCCTGCAACAGTACGGGAGGTGTTCTGA‑TGTCTGACTACCTGAAAGTGCTGCAAGCAATCAAAAGt                                                                                   <  1:103199/298‑1 (MQ=255)`  
                                                                                                                                                                                                                           `aCCGCAAAGATGTCGTCTGACGTTCAGGCAATCTTAGAGAAAGATATGCTGCATCTATGTAAGCAGGTCGGCTAAGGGGCGATTGTCCCCAATGGTAAAAAGAAGGAAATGATGGTCCAGTTCCTGAAACACGGTATGGAAGGAGGGATGACATTCGGAGGACGTACATCATTTCGTGAGGCCATTAAGGACATGCACGAAGAGTATGCAGATAAGGACTCTTTCAAAAAAGCTACTGCAACAGTACGGGAGGTGTTCTGA‑TGTCTGATTACCTGAAAGGGCTGCAAGCAATGAAAAGtag                                                                                 >  1:291043/1‑299 (MQ=255)`  
                                                                                                                                                                                                                                      `gtcgtcTGACGTTCAGGCAATCTTAGAGAAAGATATGCTGCATCTACGTAAGCAGGTCGGCTCAGGTGCGATTGTCCCCAATGGTAAACAGAAGGAAATGATTGTCCAGTTCCTGACACACGGTATGGAAGGATTGATGACATTCGTAGTACGTACATCATTTCGTGAGGCCATTAAGGACATGCACGAAGAGTATGCAGATAAGGACTCTTTCAAACAATCTCCTGCAACAGTACGGGAGGTGTTCTGA‑TGTCTGACTACCTGAAAGTGCTACAAGCAATCAAAAGTTGCCCTAAGACtt                                                                      >  1:274363/1‑301 (MQ=255)`  
                                                                                                                                                                                                                                              `aCGTTCAGGCAATCTTAGAGAAAGATATGCTGCATCTATGTAAGCAGGTCGGCTCAGGTGCGATTGTCCCCAATGGTAAACAGAAGGAAATGATTGTCCAGTTCCTGACACACGGTATGGAAGGATTGATGACATTCGTAGTACGTACATCATTTCGTGAGGCCATTAAGGACATGCACGAAGAGTATGCAGATAAGGACTCTTTCAAACAATCTCCTGCAACAGTACGGGAGGTGTTCTGA‑TGTCTGACTACCTGAAAGTGCTGCAAGCAATCAAAAGTTGCCCTAAGACTTTCCAGTcc                                                              <  1:11083/301‑1 (MQ=255)`  
                                                                                                                                                                                                                                                        `aaTCTTAGAGAAAAATATGCTGCATCTATGTAAGCAGGTCGGCTCAGGTGCGATTGTCCCCAATGGTGAACAGAAGGAAATGATTGTCCAGTTCCTGACACACGGTATGGAAGGATTGATGACATTCGTAGTACGTACATCATTTCGAGAGGCCATTAAGGAAATGCACGAAGAGTATGCAGATAAGGACTATTTCAAACAATATCCTGCAACAGTACGGGAGGTGTACTGA‑TGTCTGACTACCTGAAAGTGCTGAAAGCAATCAAAAGTTGCCCTAAGACTTTCCAGACCAACTATGTAc                                                    <  1:24556/301‑1 (MQ=255)`  
                                                                                                                                                                                                                                                         `aTCTTAGAGAAAGATATGCTGCATCTATGTAAGCAGGTCGGCTCAGGTGCGATTGTCCCCAATGGTAAACAGAAGGAAATGATTGTCCAGTTCCTGACACACGGTATGGAAGGATTGATGACATTCGTAGTACGTACATCATTTCGTGAGGCCATTAAGGACATGCACGAAGAGTATGCAGATAAGGACTCTTTCAAACAATCTCCTGCAACAGTACGGGAGGTGTTCTGA‑TGTCTGACTACCTGAAAGTGCTGCAAGCAATCAAAAGTTGCCCTAAGACTTTCCAGTCCAACTATGTACg                                                   >  1:224249/1‑301 (MQ=255)`  
                                                                                                                                                                                                                                                             `agagacaggATATGCTGCATCTATGTAAGCAGGTCGGCTCAGGTGCGATTGTCCCCAATGGTAAACAGAAGGAAATGATTGTCCAGTTCCTGACACACGGTATGGAAGGATTGATGACATTCGTAGTACGTACATCATTTCGTGAGGCCATTAAGGACATGCACGAAGAGTATGCAGATAAGGACTCTTTCAAACAATCTCCTGCAACAGTACGGGAGGTGTTCTGA‑TGTCTGACTACCTGAAAGTGCTGCAAGCAATCAAAAGTTGCCCTAAGACTTTCCAGTCCAACTATGTACGGAAc                                               <  1:240370/293‑1 (MQ=255)`  
                                                                                                                                                                                                                                                              `agagAAAGATATGCTGCATCTATGTAAGCAGGTCGGCTCAGGTGCGATTGTCCCCAATGGTAAACAGAAGGAAATGATTGTCCAGTTCCTGACACACGGTATGGAAGGATTGATGACATTCGTAGTACGTACATCATTTCGTGAGGCCATTAAGGACATGCACGAAGAGTATGCAGATAAGGACTCTTTCAAACAATCTCCTGCAACAGTACGGGAGGTGTTCTGA‑TGTCTGACTACCTGAAAGTGCTGCAAGCAATCAAAAGTTGCCCTAAGACTTTCCAGTCCAACTATGTACGGAACa                                              >  1:145030/1‑301 (MQ=255)`  
                                                                                                                                                                                                                                                                  `agaaaggtcaggtaTCTATGTAGGCATGTCGGGTCAGGTGCGTTTTTCCCTAATGGTAAACAGAATGATATGATTGTCCAGTTCCTAGCAAACGTGATGACAGGATTGATGAAATTCGTAGTTATTACATCGTTTCTTGAGGGCAGTAATGACATGAACGAATAGTATGCAGATATGTACTCTTTCAAATAATCTCCTGTAACAGTATGGGGGGTGTTCTGA‑TGTCTGACTACCTGAAAGTGCTGCAAGCAATAAAAAGTTGCCCTAAGACTTTCCAGTCCAACTATGTACGGAACAATGc                                          <  1:213669/288‑1 (MQ=255)`  
                                                                                                                                                                                                                                                                     `gATATGCTGCATCTATGTAAGCAGGTCGGCTCAGGTGCGATTGTCACCAATGGTAAACAGAAGGAAATGATTGGCCAGTTCCTGACACACGGTATGGAAGGATTGATGACATTCGTAGTACGTACATCATTTCGTGAGGCCCTTAAGGACATTCACGAAGAGTATGCAGATAAGGACTCTTTCAAACAATCACCTGCAACAGTACGGGCGGTGTTCTTA‑TGTCTGACTAACTGACAGTGATGCAAGCAATCAAAAGTTGACCTAAGAATTTCAAGTACAACAATGTACGGAACAATGagag                                       >  1:347409/1‑301 (MQ=255)`  
                                                                                                                                                                                                                                                                      `cagttctgcATCTATGTAAGCAGGTCGGCTCAGGTGCGATTGTCCCCAATGGTAAACAGAAGGAAATGATAGTCCAGTTCCTGACACACGGTATGGAAGGATTGATGACATTCGTAGTACGTACATCATTTCGTGAGGCCATTAAGGACATGCACGAAGAGTATGCAGATAAGTACTCTTTCAAACAATCTCCTGCAACAGTACGGGAGGTGTTCTGA‑TGTCTGACTACCTGAAAGTGCTGCAAGCAATCAAAAGTTGCCCTAAGACTTTCCAGTCCAACTATGTACGGAACAATGCGAGc                                      <  1:44212/296‑1 (MQ=255)`  
                                                                                                                                                                                                                                                                      `acaggctgcATATATGTAAGCAGGTCGGCTCAGGTGCGATTGTCCCCAATGGTAAACAGAAGGAAATGATTGTCCAGTTCCTGACACACGGTATGGAAGGATTGATGACATTCGTAGTACGTACATCATTTCGTGAGGCCATTAAGGACATGCACGAAGAGTATGCAGATAAGGACTCTTTCAAACAATCTCCTGCAACAGTACGGGAGGTGTTCTGA‑TGTCTGACTACCTGAAAGTGCTGCAAGCAATCAAAAGTTGCCCTAAGACTTTCCAGTCCAACTATGTACGGAACAATGCGAGc                                      <  1:100533/297‑1 (MQ=255)`  
                                                                                                                                                                                                                                                                             `gcATCTATGTAAGCAGGTCGGCTCAGGTGCGATTGTCCCCAATGGTAAACAGAAGGAAATGATTGTCCAGTTCCTGACACACGGTATGGAAGGATTGATGACATTCGTAGTACGTACATCATTTCGTGAGGCCATTAAGGACATGCACGAAGAGTATGCAGATAAGGACTCTTTCAAACAATCTCCTGCAACAGTACGGGAGGTGTTCTGA‑TGTCTGCCTACCTGAAAGTGCTGCAAGCAATCAAAAGTTGCCCTAAGACTTTCCAGTCCAACTATGTACGGAACAATGCGAGCCTCGTAg                               >  1:286350/1‑301 (MQ=255)`  
                                                                                                                                                                                                                                                                             `gcATCTATGTAAGCAGGTCGGCTCAGGTGCGATTGTCCCCAATGGTAAACAGAAGGAAATGATTGTCCAGTTCCTGACACACGGTATGGAAGGATTGATGACATTCGTAGTACGTACATCATTTCGTGAGGCCATTAAGGACATGCACGAAGAGTATGCAGATAAGGACTCTTTCAAACAATCTCCTGCAACAGTACGGGAGGTGTTCTGA‑TGTCTGACTACCTGAAAGTGTTGCAAGCAATCAAAAGTTGCCCTAAGACTTTCCAGTCCAACTATGTACGGAACAATGCGAGCCTCGTAg                               >  1:115398/1‑301 (MQ=255)`  
                                                                                                                                                                                                                                                                                `tCTATGTAAGCAGGTCGGCTCAGGTGCGATTGTCCCCAATGGTAAACAGAAGGAAATGATTGTCCAGTTCCTGACACACGGTATGGAAGGATTGATGACATTCGTAGTACGTACATCATTTCGTGAGGCCATTAAGGACATGCACGAAGAGTATGCAGATAAGGACTCTTTCAAACAAACTCCTGCAACAGTACGGGAGGTGTTCTGA‑TGTCTGACTACCTGAAAGTGCTGCAAGCAATCAAAAGTTGCCCTAAGACTTTCCAGTCCAACTATGTACGGAACAATGCGAGCCTCGTAGCgg                            >  1:6981/1‑301 (MQ=255)`  
                                                                                                                                                                                                                                                                                  `aatttAAGCAGGGAGGCTCAGGTGCGATTGTCCCCAAAGGTAAACAGAAGGAAATGATTGTCCAGTTCATGTCAAACGGGATGGAAGGATTGATGACATTCGGAGTACGTACATCATTTCGTGAGGCCATTAAGGACATGCAAGAAGAGTATGCAGATAATGACTCTTTCAAACAATCTCCTGCAACAGTACGGGAGGTGTTCTGA‑TGTCTGACTACCTGAAAGTGCTGCAAGCAATCAAAAGTTGCCCTAAGACTTTCCAGTCCAACTATGTACGGAACAATGCGAGCCTCGTAGCGGAg                          <  1:73282/297‑1 (MQ=255)`  
                                                                                                                                                                                                                                                                                       `aaGCAGGTCGGCTCAGGTGCGATTGTCCCCAATGGTAAACAGAAGGAAATGATTGTCCAGTTCCTGACACACGGTATGGAAGGATTGATGACATTCGTAGTACGTACATCATTTCGTGAGGCCATTAAGGACATGCACGAAGAGTATGCAGATAAGGACTCTTTCAAACAATCTCCTGCAACAGTACGGGAGGTGTTCTGA‑TGTCTGACTACCTGAAAGTGCTGCAAGCAATCAAAAGTTGCCCTAAGACTTTCCAGTCCAACTATGTACGGactgtctcttatacccctctccgagccca                     >  1:60688/1‑273 (MQ=255)`  
                                                                                                                                                                                                                                                                                            `ggTCGGCTCAGGTGCGATTGTCCCCAATGGTAAACAGAAGGAAATGATTGTCCAGTTCCTGACACACGGTATGGAAGGATTGATGACATTCGTAGTACGTACATCATTTCGTGAGGCCATTAAGGACATGCACGAAGAGTATGCAGATAAGGACTCTTTCAAACAATCTCCTGCAACAGTACGGGAGGTGTTCTGA‑TGTCTGACTACCTGAAAGTGCTGCAAGCAATCAAAAGTTGCCCTAAGACTTTCCAGTCCAACTATGTACGGAACAATGCGAGCCTCGTAGCGGAGGCCGCTTccc                <  1:259282/301‑1 (MQ=255)`  
                                                                                                                                                                                                                                                                                            `acacttaacAGGTGCGATTGTCCCCAATGGTAAACAGAAGGAAATGATTGTCCAGTTCCTGACACACGGTATGGAAGGATTGATGACATTCGTAGTACGTACATCATTTCGTGAGGCCATTAAGGACATGCACGAAGAGTATGCAGATAAGGACTCTTTCAAACAATCTCCTGCAACAGTACGGGAGGTGTTCTGA‑TGTCTGACTACCTGAAAGTGCTGCAAGCAATCAAAAGTTGCCCTAAGACTTTCCAGTCCAACTATGTACGGAACAATGCGAGCCTCGTAGCGGAGGCCGCTTccc                >  1:119339/9‑301 (MQ=255)`  
                                                                                                                                                                                                                                                                                               `cttaacAGGTGCGATTGTCCCCAATGGTAAACAGAAGGAAATGATTGTCCAGTTCCTGACACACGGTATGGAAGGATTGATGACATTCGTAGTACGTACATCATTTCGTGAGGCCATTAAGGACATTCACGAAGAGTATGCAGATAAGGAATCTTTCAAACACTCTCCTGCAACAGTACGGGAGGTGTTCTGA‑TGTCTGTCTAACTGAAAGTGCTTCAAGCAATCAAAAGTTTCCCTAAGACTTTCCAGTACCACTATGTCCGGAACAATGCCACCCTTCTCACGGAGGCCGCTTACCGTg             >  1:330613/6‑301 (MQ=255)`  
                                                                                                                                                                                                                                                                                                       `gTGCGATTGTCCCCAATGGTAAACAGAAGGAAATGATTGTCCAGTTCCTGACACACGGTATGGAAGGATTGATGACATTCGTAGTACGTACATCATTTCGTGAGGCCATTAAGGACATGCACGAAGAGTATGCAGATAAGGACTCTTTCAAACAATCTCCTGCAACAGTACGGGAGGTGTTCTGA‑TGTCTGACTACCTGAAAGTGCTGCAAGCAATCAAAAGTTGCCCTAAGACTTTCCAGTCCAACTATGTACGGAACAATGCGAGCCTCGTAGCGGAGGCCGCTTCCCGTGGTcacact     >  1:305310/1‑299 (MQ=255)`  
                                                                                                                                                                                                                                                                                                        `tGCGATTGTCCCCAATGGTAAACAGAAGGAAATGATTGTCCAGTTACAGACACACGGTATGGAAGGATTGATGACATTCGTAGTACGTACATCATTTCGTGAGGCCATTAAGGACATGCACGAAGAGTATGCAGATATGGACTCTTTCAAACAATCTCCTGCAACAGTACGTGAGGTGTTCTGA‑TGTCTGACTACCTGAAAGTGCTGCAAGCAATCAAAAGTTGCCCTAAGACTTTCCAGTCCAACTATGTACGGAACAATGCGAGCCTCGTAGCGGAGGCCGCTTCCCGTGGTCACAtct    <  1:88412/301‑1 (MQ=255)`  

                                                                                                                                                                                                                                                                                                           `|`                                                                                                                                                                                                                                                                                                              
`TGCCAAGCAGGTTAGGCTAGGTGGATGCGGTAGGTCCGAATATAAGGATGGAGACTTTGACATTCTTGCGGTTGTGGTTGACGAAGATGTGCTTATTTTCACATGGGACGAAGTAAAAGGTAAGACATCCATGTGTGTCGGCAAGAGAAACAAAGGCATAAAACTATAGGAGAAATTATTATGGCTATGACAAAGAAATTTAAAGTGTCCTTCGACGTTACCGCAAAGATGTCGTCTGACGTTCAGGCAATCTTAGAGAAAGATATGCTGCATCTATGTAAGCAGGTCGGCTCAGGTGCGATTGTCCCCAATGGTAAACAGAAGGAAATGATTGTCCAGTTCCTGACACACGGTATGGAAGGATTGATGACATTCGTAGTACGTACATCATTTCGTGAGGCCATTAAGGACATGCACGAAGAGTATGCAGATAAGGACTCTTTCAAACAATCTCCTGCAACAGTACGGGAGGTGTTCTGA‑TGTCTGACTACCTGAAAGTGCTGCAAGCAATCAAAAGTTGCCCTAAGACTTTCCAGTCCAACTATGTACGGAACAATGCGAGCCTCGTAGCGGAGGCCGCTTCCCGTGGTCACATCTCG  >  NC_001604.1/16671‑17269`

  

Alignment Legend

Aligned base mismatch/match (shaded by quality score): `ATCG/ATCG` `< 3 ≤` `ATCG/ATCG` `< 11 ≤` `ATCG/ATCG` `< 23 ≤` `ATCG/ATCG` `< 35 ≤` `ATCG/ATCG` `< 38 ≤` `ATCG/ATCG`

Unaligned base: `atcg`    Masked matching base: `atcg`    Alignment gap: `‑`    Deleted base: `‑`

Reads not counted as support for junction

read\_name Not counted due to insufficient overlap past the breakpoint.

read\_name Not counted due to not crossing MOB target site duplication.
